# Supplementary material for: Nonlinear transport and radio frequency rectification in BiTeBr at room temperature
Source: Nat Commun. 2024 Jan 4;15:245. doi: 10.1038/s41467-023-44439-w (PMC10764878; doi:10.1038/s41467-023-44439-w)
Supplement: Supplementary file 1 — supplementary information [file 41467_2023_44439_MOESM1_ESM.pdf]

Supplementary information

Nonlinear Transport and Radio Frequency Rectification in BiTeBr at Room Temperature

Xiu Fang Lu[1,#], Cheng-Ping Zhang[2,#], Naizhou Wang[3], Dan Zhao[4], Xin Zhou[1], Weibo Gao[3], Xian Hui Chen[4], K. T. Law[2*], and Kian Ping Loh[1*]

[1]Department of Chemistry, National University of Singapore, Singapore 117543, Singapore.

[2]Department of Physics, Hong Kong University of Science and Technology, Hong Kong, China.

[3]Division of Physics and Applied Physics, School of Physical and Mathematical Sciences, Nanyang Technological University, Singapore 637371, Singapore.

[4]Department of Physics and Hefei National Laboratory for Physical Science at Microscale, University of Science and Technology of China, Hefei, Anhui 230026, P. R. China.

[#]These authors contributed equally: Xiu Fang Lu, Cheng-Ping Zhang

E-mail: phlaw@ust.hk, chmlohkp@nus.edu.sg

This file includes:

S1. Theory of second-order response induced by skew scattering in BiTeBr.

S2. Orientation of exfoliated BiTeBr flake

S3. Excluding other extrinsic origins for nonlinear response

S4. Nonlinear transport in different thickness device

S5. Symmetry-dependent nonlinear response

S6. Fitting the experimental nonlinear data to the theoretically derived equation

S7. Additional wireless radiofrequency rectification data

S1. Theory of second-order response induced by skew scattering in BiTeBr

General theory

The Boltzmann equation reads

$$\frac{\partial f}{\partial t} - \frac{e\boldsymbol{E}}{\hbar} \cdot \nabla_{\boldsymbol{k}} f = -\int_{\boldsymbol{k}'} [W_{\boldsymbol{k}'\boldsymbol{k}} f(\boldsymbol{k}) - W_{\boldsymbol{k}\boldsymbol{k}'} f(\boldsymbol{k}')], \tag{1}$$

where $f(\boldsymbol{k})$ is the Fermi distribution function in the nonequilibrium state, $W_{\boldsymbol{k}\boldsymbol{k}'}$ is the scattering rate due to impurity. The scattering rate can be decomposed into the symmetric part $W^S_{\boldsymbol{k}\boldsymbol{k}'} = (W_{\boldsymbol{k}\boldsymbol{k}'} + W_{\boldsymbol{k}'\boldsymbol{k}})/2$ and anti-symmetric part $W^A_{\boldsymbol{k}\boldsymbol{k}'} = (W_{\boldsymbol{k}\boldsymbol{k}'} - W_{\boldsymbol{k}'\boldsymbol{k}})/2$. Following standard perturbation theory, they can be derived as[1]

$$W^S_{\boldsymbol{k}\boldsymbol{k}'} = 2\pi < |V_{\boldsymbol{k}\boldsymbol{k}'}|^2 >_{dis} \delta(\epsilon_{\boldsymbol{k}} - \epsilon_{\boldsymbol{k}'}), \tag{2}$$

$$W^A_{\boldsymbol{k}\boldsymbol{k}'} = -(2\pi)^2 \int_{\boldsymbol{k}''} \mathrm{Im} < V_{\boldsymbol{k}\boldsymbol{k}'} V_{\boldsymbol{k}''\boldsymbol{k}} V_{\boldsymbol{k}'\boldsymbol{k}''} >_{dis} \delta(\epsilon_{\boldsymbol{k}} - \epsilon_{\boldsymbol{k}'}) \delta(\epsilon_{\boldsymbol{k}} - \epsilon_{\boldsymbol{k}''}), \tag{3}$$

where $<>_{dis}$ implies impurity average, and $V_{\boldsymbol{k}\boldsymbol{k}'} =< \boldsymbol{k}|\hat{V}|\boldsymbol{k}' >$ is the matrix element of the scattering potential.

In order to solve the above Boltzmann equation, we can decompose the distribution function f up to second-order

$$f = f_0 + f_1^S + f_1^A + f_2^S + f_2^A, \tag{4}$$

where f_0 is the equilibrium distribution function, the subscript and the superscript indicate the orders of the electric field E and the skew scattering rate W^A respectively. In the next section, we show how the Boltzmann equation can be solved order by order.

Once the distribution function is obtained, the current response is

$$\boldsymbol{j} = -e \int_{\boldsymbol{k}} \boldsymbol{v}(\boldsymbol{k}) f(\boldsymbol{k}), \tag{5}$$

where

$$\boldsymbol{v}(\boldsymbol{k}) = \frac{1}{\hbar} \cdot \nabla_{\boldsymbol{k}} \epsilon_{\boldsymbol{k}} + \frac{e}{\hbar} \boldsymbol{E} \times \boldsymbol{\Omega}(\boldsymbol{k}) \tag{6}$$

is contributed by both the group velocity and the anomalous velocity. Specifically, the skew scattering contribution to the second-order response is

$$\boldsymbol{j} = -\frac{e}{\hbar} \int_{\boldsymbol{k}} \nabla_{\boldsymbol{k}} \epsilon_{\boldsymbol{k}} f_2^A. \tag{7}$$

Solving the Boltzmann equation

Here we consider the situation $\omega\tau \ll 1$, which can usually be satisfied when the frequency is much lower than THz, given the scattering time $\tau \lesssim 1$ ps. In this case, the first term $\frac{\partial f}{\partial t}$ in the Boltzmann equation can be ignored. Substituting the distribution function Eq.4 into the Boltzmann equation Eq.1, we get

$$f_1^S(\boldsymbol{k}) = \frac{e\tau}{\hbar} E_a \partial_a f_0, \tag{8}$$

$$f_1^A(\boldsymbol{k}) = \tau \int_{\boldsymbol{k}'} W_{\boldsymbol{k}\boldsymbol{k}'}^A f_1^S(\boldsymbol{k}'), \tag{9}$$

$$f_2^S(\boldsymbol{k}) = \frac{e^2\tau^2}{\hbar^2} E_a E_b \partial_a \partial_b f_0, \tag{10}$$

$$f_2^A(\boldsymbol{k}) = \tau \int_{\boldsymbol{k}'} W_{\boldsymbol{k}\boldsymbol{k}'}^A f_2^S(\boldsymbol{k}') + \frac{e\tau}{\hbar} E_b \partial_b f_1^A, \tag{11}$$

where $\partial_a \equiv \partial/\partial_{k_a}$, $a, b = x, y, z$ are spatial indices, and the scattering time is defined as $\int_{\boldsymbol{k}'} W_{\boldsymbol{k}\boldsymbol{k}'}^S [f(\boldsymbol{k}) - f(\boldsymbol{k}')] \equiv \frac{1}{\tau} f(\boldsymbol{k})$.

Note that the scattering time is yet to be solved self-consistently, with the explicit form of the Hamiltonian. In the following sections, we will solve the scattering time for the 2D Rashba model.

Skew scattering in Rashba bands

We consider the Hamiltonian of 2D Rashba bands

$$\hat{H}_0 = \epsilon_0(k) + \alpha_R\left(k_y\sigma_x - k_x\sigma_y\right) + \frac{\lambda}{2}(k_+^3 + k_-^3)\sigma_z, \tag{12}$$

where we have added a third-order warping term. The energy dispersion is $\epsilon_\pm(\boldsymbol{k}) = \epsilon_0(k) \pm \sqrt{\alpha_R^2 k^2 + \lambda^2 k^6 \cos^2 3\theta_{\boldsymbol{k}}}$, and the Berry curvature $\Omega_\pm(\boldsymbol{k}) = \mp\frac{\lambda}{\alpha_R}\cos 3\theta_{\boldsymbol{k}} + \mathcal{O}(\lambda^2)$, with $\theta_{\boldsymbol{k}} = \arg \boldsymbol{k}$. The *d*-vector can be parameterized as

$$\begin{aligned} \boldsymbol{d}(\boldsymbol{k}) &= \left[\alpha_R k_y, -\alpha_R k_x, \frac{\lambda}{2}(k_+^3 + k_-^3)\right] \\ &\equiv |\boldsymbol{d}(\boldsymbol{k})|[\sin\phi_{\boldsymbol{k}} \sin\theta_{\boldsymbol{k}}, -\sin\phi_{\boldsymbol{k}}\cos\theta_{\boldsymbol{k}}, \cos\phi_{\boldsymbol{k}}], \end{aligned} \tag{13}$$

and the wave functions of the upper and lower bands are

$$|\boldsymbol{k},+\rangle=\left(\cos\frac{\phi_k}{2},-i\sin\frac{\phi_k}{2}e^{i\theta_k}\right)^{\mathrm{T}}, \tag{14}$$

$$|\boldsymbol{k},-\rangle=\left(\sin\frac{\phi_k}{2}e^{-i\theta_k},-i\cos\frac{\phi_k}{2}\right)^{\mathrm{T}}. \tag{15}$$

Therefore, the form factors can be calculated as

$\langle\boldsymbol{k}|\boldsymbol{k}'\rangle_+=\langle\boldsymbol{k}'|\boldsymbol{k}\rangle_-$

$$=\frac{1}{2}\left[1+e^{-i(\theta_k-\theta_{k'})}\right]+\frac{\lambda k^2}{4\alpha_R}(\cos 3\theta_{\boldsymbol{k}}+\cos 3\theta_{\boldsymbol{k}'})\left[1-e^{-i(\theta_k-\theta_{k'})}\right]+\mathcal{O}(\lambda^2). \tag{16}$$

Then, we can consider the screened Coulomb interaction[2] $V(q)=\frac{2\pi\alpha\alpha_R}{q+q_{TF}}$, where α is the dimensionless coupling constant. The matrix element of the Coulomb interaction is $V_{\boldsymbol{k}\boldsymbol{k}'}=\frac{2\pi\alpha\alpha_R}{|\boldsymbol{k}-\boldsymbol{k}'|+q_{TF}}\langle\boldsymbol{k}|\boldsymbol{k}'\rangle$, and therefore we get the scattering rate

$$W_{\boldsymbol{k}\boldsymbol{k}'}^{S,\pm}=\frac{4\pi^3 n_i\alpha^2\alpha_R^2}{(|\boldsymbol{k}-\boldsymbol{k}'|+q_{TF})^2}[1+\cos(\theta_{\boldsymbol{k}}-\theta_{\boldsymbol{k}'})]\delta(\epsilon_{\boldsymbol{k}}-\epsilon_{\boldsymbol{k}'})+\mathcal{O}(\lambda), \tag{17}$$

which leads to the scattering time $\tau\approx(\int_{\boldsymbol{k}'}W_{\boldsymbol{k}\boldsymbol{k}'}^{S})^{-1}\approx\frac{\hbar V_F k_F}{\pi^2 n_i\alpha^2\alpha_R^2}$, where $V_F=\frac{1}{\hbar}\left|\frac{\partial\epsilon_k}{\partial k}\right|_{k=k_F}$ is the Fermi velocity.

Considering the symmetry properties of the antisymmetric scattering rate $W_{\boldsymbol{k}\boldsymbol{k}'}^{A}=-W_{\boldsymbol{k}'\boldsymbol{k}}^{A}$, $W_{\hat{C}_3\boldsymbol{k},\hat{C}_3\boldsymbol{k}'}^{A}=W_{\boldsymbol{k}\boldsymbol{k}'}^{A}$, $W_{\hat{M}_x\boldsymbol{k},\hat{M}_x\boldsymbol{k}'}^{A}=W_{\boldsymbol{k}\boldsymbol{k}'}^{A}$, the leading-order shall take the form $W_{\boldsymbol{k}\boldsymbol{k}'}^{A}\sim(\cos 3\theta_{\boldsymbol{k}}+\cos 3\theta_{\boldsymbol{k}'})\sin(\theta_{\boldsymbol{k}}-\theta_{\boldsymbol{k}'})$. Therefore, the antisymmetric scattering rate can be approximated as

$$W_{\boldsymbol{k}\boldsymbol{k}'}^{A,\pm}=\mp\frac{\pi^4 c_0 n_i\alpha^3\alpha_R^2\lambda}{2\hbar V_F}\frac{\delta(\epsilon_{\boldsymbol{k}}-\epsilon_{\boldsymbol{k}'})}{\left|\sin\frac{\theta_k-\theta_{k'}}{2}\right|+\frac{q_{TF}}{2k}}(\cos 3\theta_{\boldsymbol{k}}+\cos 3\theta_{\boldsymbol{k}'})\sin(\theta_{\boldsymbol{k}}-\theta_{\boldsymbol{k}'})+\mathcal{O}(\lambda^2), \tag{18}$$

where $c_0\approx 1.15$ when $\frac{q_{TF}}{2k_F}\ll 1$.

Nonlinear response induced by skew scattering

Next, we consider a current applied along the *x*-direction and calculate the nonlinear response in both the *x*- and *y*-directions induced by skew scattering.

First of all, we study the simple case when $\epsilon_0(k)=0$ at temperature $T=0$. In this

case, we can obtain an analytical result, which provides insight into the nonlinear response.

Substituting the scattering rate into Eqs. 8-11, we get the second-order conductivity

$$\sigma_{xxx} = 0, \tag{19}$$

$$\sigma_{yxx} \simeq \pm\mathrm{sgn}(\lambda)\frac{e^3\alpha_R\tau^3}{\hbar^3\tilde{\tau}}, \tag{20}$$

where the skew scattering time $\tilde{\tau} = 16/(\pi^2 c_1 n_i \alpha^3 |\lambda| k_F)$, with $c_1 \simeq 1.25$. From this result, we can conclude several important features.

(i) The second-order nonlinear response induced by skew scattering is proportional to τ^3, which is consistent with the scaling behaviour in the experiment.

(ii) The contributions from upper and lower Rashba bands have opposite signs, which will lead to cancellation when the temperature is higher than the Rashba splitting energy.

(iii) The scattering time $\tau \propto k_F$ and the skew scattering time $\tilde{\tau} \propto k_F^{-1}$, which implies that the nonlinear conductivity $\sigma^{(2)} \propto k_F^4$. This will lead to a rapid growth of the nonlinear response when the chemical potential is gated from the band edge.

The effect of the parabolic background

Now, we add back the parabolic background $\epsilon_0(k) = tk^2$ and study the nonlinear response. We plot the band structure in Figure S1a for reference. The Rashba band minimum is at $k_0 = \frac{\alpha_R}{2t}$, with the energy $E = -\epsilon_R \equiv -\frac{\alpha_R^2}{4t}$. The nonlinear response is mainly contributed by the lower band, as plotted in Figure S1b. It increases fast as the chemical potential is gated from the band edge, which is consistent with the experiment. However, we would like to point out that our $\boldsymbol{k} \cdot \boldsymbol{p}$ model only applies to the band edge. The Rashba spin-orbit coupling will eventually diminish when k_F is far away from the high symmetry point, which will lead to the decrease of the nonlinear response when the chemical potential is further gated away from the band edge.

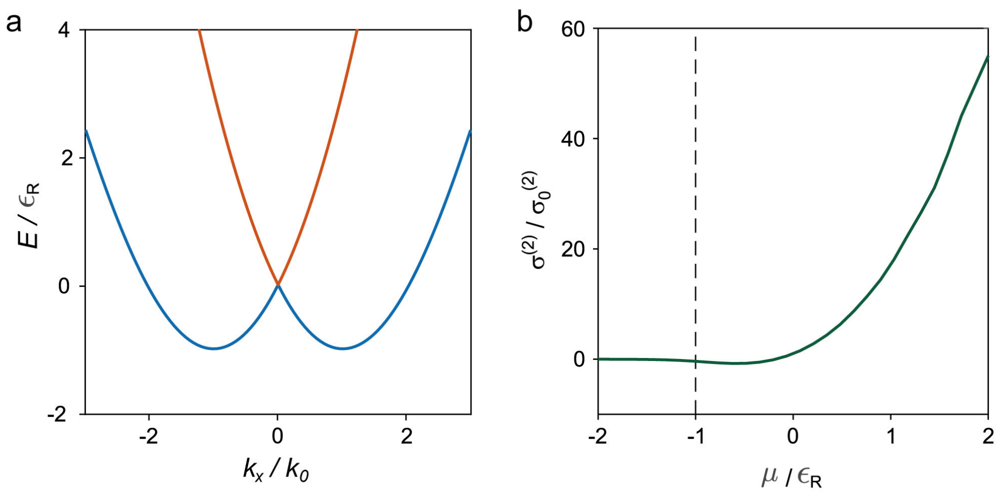

Supplementary Figure S1 | **a**, The band structure of the Rashba model. **b**, Calculated gate dependence of the nonlinear conductivity, where the black dashed line indicates the band edge. Here, $k_0 = \frac{\alpha_R}{2t}$, $\epsilon_R = \frac{\alpha_R^2}{4t}$, and we have adopted the parameter $\lambda k_0^3/\epsilon_R = 0.1$ for the skew scattering calculation. The nonlinear conductivity is normalised with $\sigma^{(2)}{}_0 = \sigma^{(2)}|_{\mu=0}$.

S2. Orientation of exfoliated BiTeBr flake

The crystal axes of exfoliated BiTeBr flakes were determined using two techniques: second harmonic generation (SHG) and scanning transmission electron microscopy (STEM). Figure S2a and b display an optical image of a BiTeBr flake exfoliated with the Al_2O_3-assisted mechanical exfoliation method and the corresponding SHG intensity at different incident laser polarized angles. The polarization of the incident laser was controlled using a half-wave plate. BiTeBr flake was aligned at 0 degrees of the half-wave plate along the direction of the red arrow. This direction was identified as the crystal axis of the BiTeBr flake[3-6].

To further confirm the crystal orientation of the BiTeBr flake, a STEM measurement was conducted on the BiTeBr device after electrical nonlinear transport measurement. The sample for STEM was prepared using focused ion beam (FIB) and milled along the current electrodes of the BiTeBr Hall bar device, as illustrated in Figure S2c. The STEM image presented in Figure S2d indicates that this crystallographic plane is the (210) plane, thus corroborating the SHG measurement result.

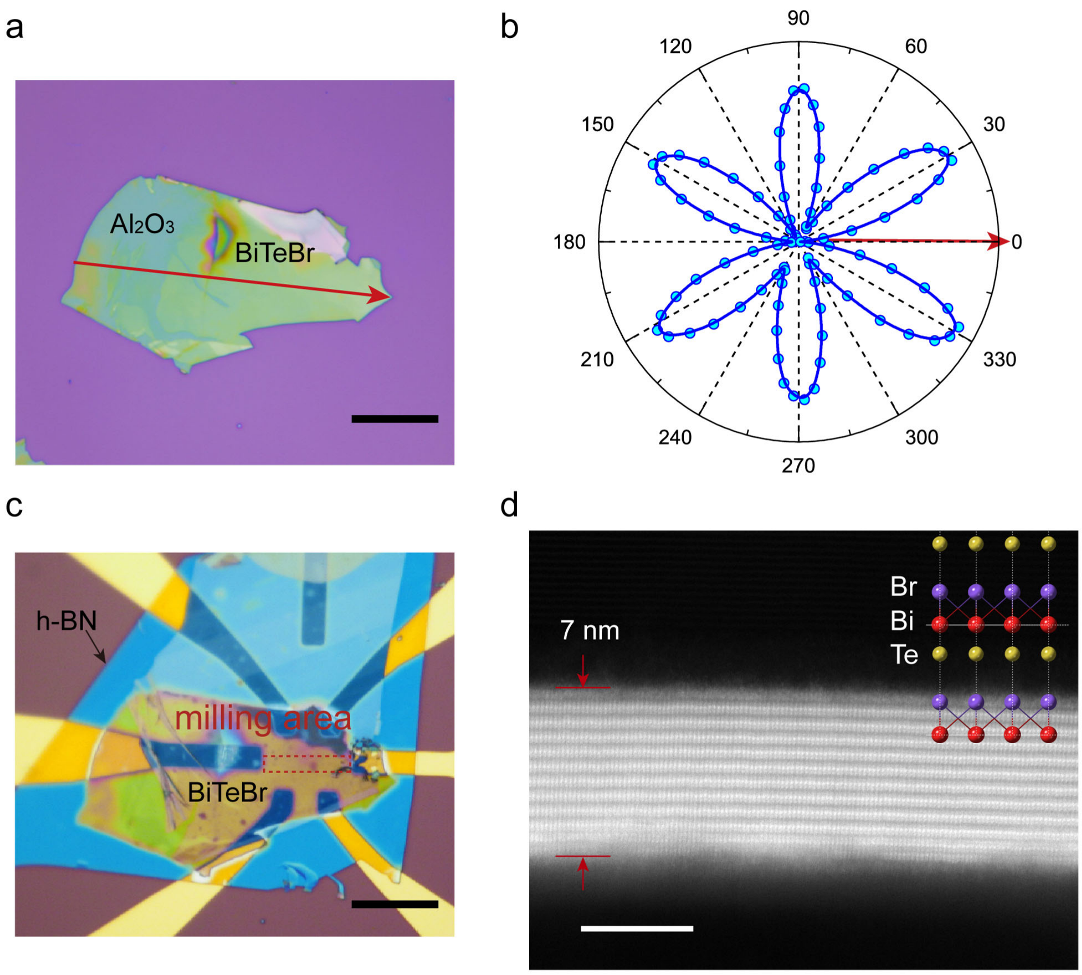

Supplementary Figure S2 | The crystal orientation of BiTeBr flake. a and **b** Optical image of BiTeBr flake exfoliated with the Al_2O_3-assisted mechanical exfoliation method (a) and the corresponding second harmonic generation (SHG) intensity at different incident laser polarized angle (b). Scale bar, 20 μm. (c) and (d) Optical image of BiTeBr Hall bar device for scanning transmission electron microscopy (STEM) measurement (c) and the corresponding cross-section STEM image (d). Scale bar in (c) and (d), 20 μm and 5 nm respectively.

S3. Excluding other extrinsic origins for nonlinear response

To exclude other extrinsic origins, like accidental diodes between the sample and contact junction, the two-terminal DC *I*-*V* relationship and nonlinear response across different pairs of contacts were conducted. The two-terminal DC *I*-*V* characteristics of 4 nm-thick BiTeBr device are presented in Figure S3, exhibiting a consistently linear behaviour across all electrodes and suggesting good ohmic contact. Figure S4 shows the nonlinear response at both longitudinal and transverse direction measured across different pairs of contacts in 4 nm device, demonstrating that the second-order nonlinear response does not rely on the specific pairs of contacts used for measurement.

The two-terminal DC *I*-*V* characteristics of 9 nm, 15 nm, and 25 nm thick device are shown in Figure S5, both of which exhibit good ohmic contact.

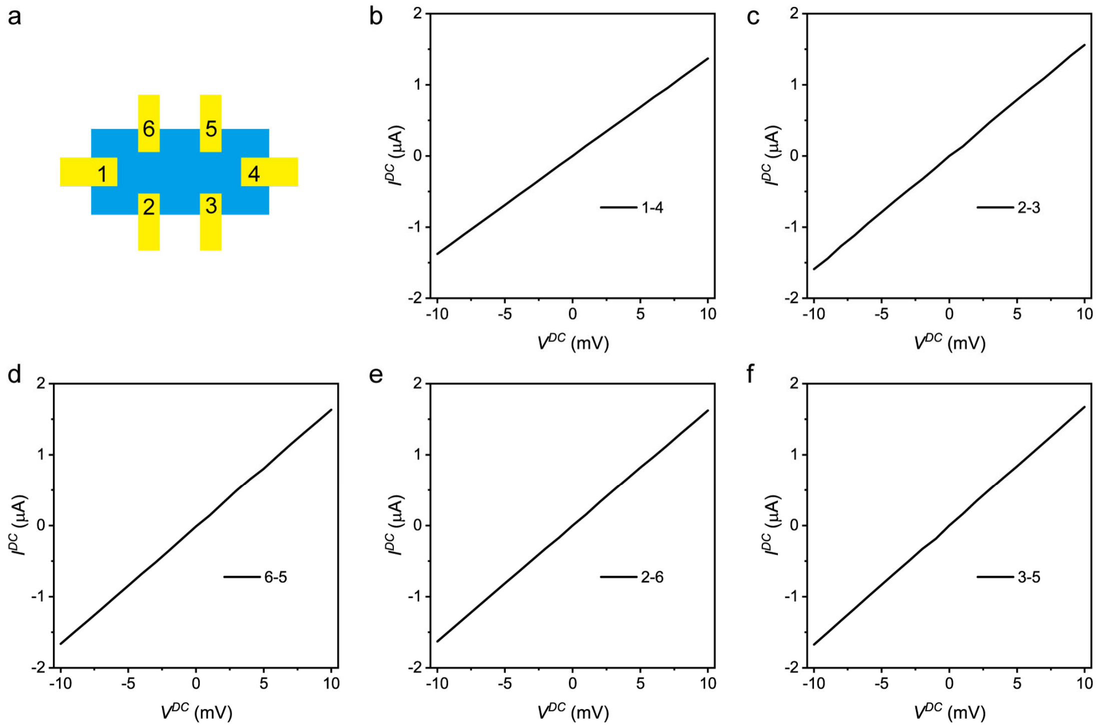

Supplementary Figure S3 | Two-terminal DC characteristics of 4 nm-thick BiTeBr device. a, Schematic illustration of 4 nm-thick BiTeBr Hall bar device with labelled electrode numbers. **b** to **f**, Two-terminal *I*-*V* characteristics for all electrodes.

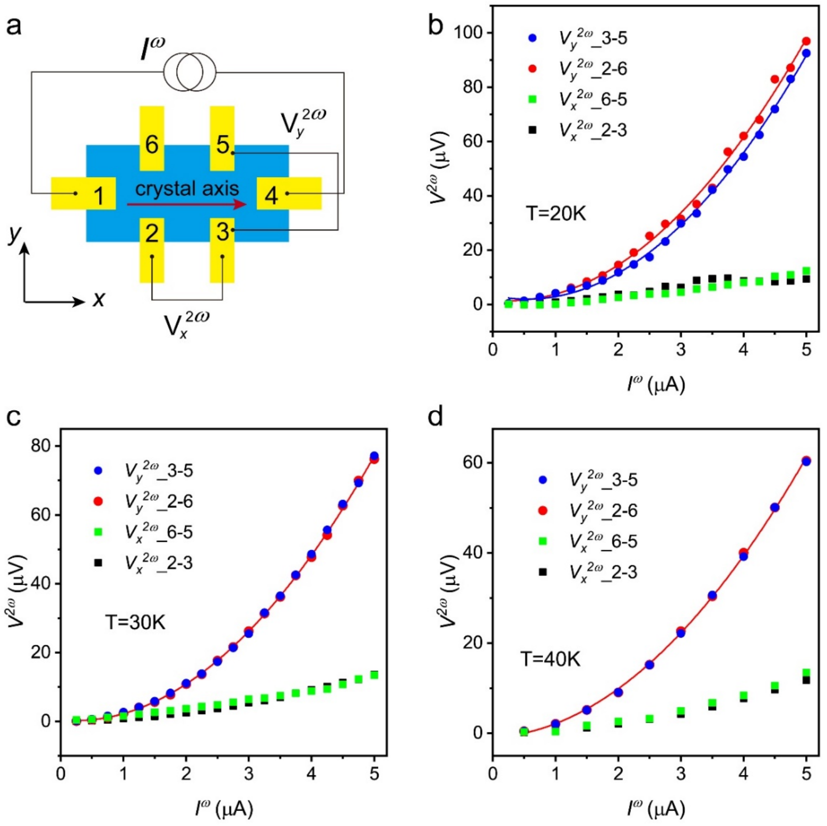

Supplementary Figure S4 | Nonlinear response measured across different pairs of contacts. **a**, Schematic illustration of 4nm-thick BiTeBr Hall bar device and the number of electrodes. **b**, **c**, and **d**, Second order longitudinal ($V_x^{2\omega}$) and transverse ($V_y^{2\omega}$) as a function of current measured across different pairs of contacts at 20K, 30K, and 40K.

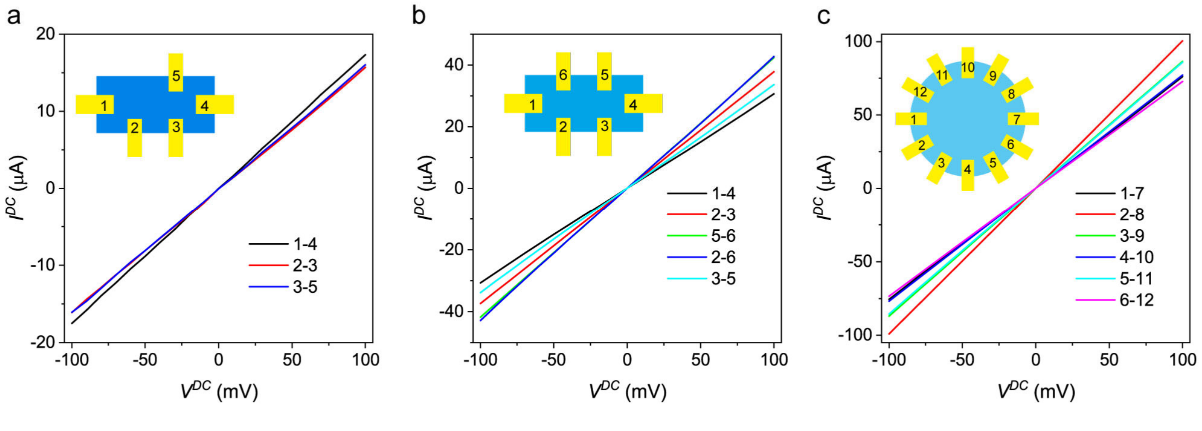

Supplementary Figure S5 | Two-terminal DC characteristics of 9 nm, 15nm, and 25 nm thick BiTeBr devices, respectively.

Figure S6 shows the second-harmonic nonlinear transverse voltage ($V_y^{2\omega}$) as a function of alternating current under different driving frequencies in the BiTeBr device measured at room temperature. No frequency dependence was observed in the frequency range from 7 to 277 Hz, excluding the spurious capacitive coupling effect.

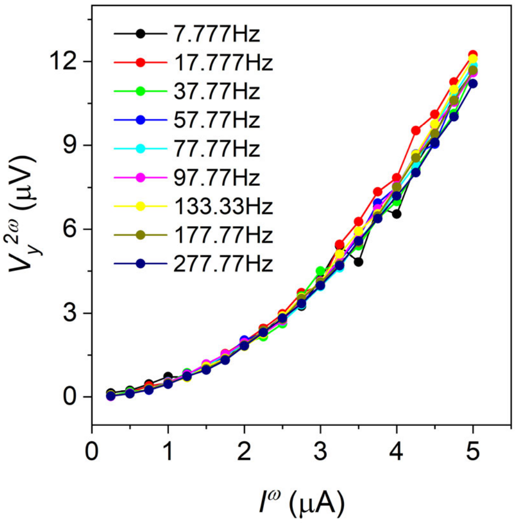

Supplementary Figure S6 | Frequency dependence of nonlinear transverse response of 22 nm thick BiTeBr device. Second-order transverse response measured at room temperature with the current along crystal axis under different driving frequencies.

S4. Nonlinear transport in different thickness BiTeBr device

To gain deeper insights into the origins of the observed nonlinear response in BiTeBr, we investigated the scaling behavior of nonlinear susceptibility $E_y^{2\omega}/(E_x)^2$ as a function of the square of conductivity σ^2 in different thickness devices. The nonlinear transport data for 9 nm, and 15 nm thick device are presented in Figure S7 and S8, revealing a pronounced nonlinear response that persists up to room temperature. The linear scaling of the nonlinear susceptibility with σ^2 allow us to extract two contributions to the second-order conductivity$\sigma_{yxx}^{(2)}$, scaling as σ^3 and σ, respectively. These contributions quantify the influence of skew scattering and side jump on the nonlinear response in our BiTeBr system.

The σ^3 and σ dependence with respect to the skew scattering and side jump

contributions to the second harmonic conductivity $\sigma_{yxx}^{(2)}$ in 4 nm, 9 nm, and 15 nm thick BiTeBr devices are shown in Figure S9. The skew scattering contribution dominates in all devices. In addition, with the increase of thickness, the side jump contribution changes its sign from negative to positive.

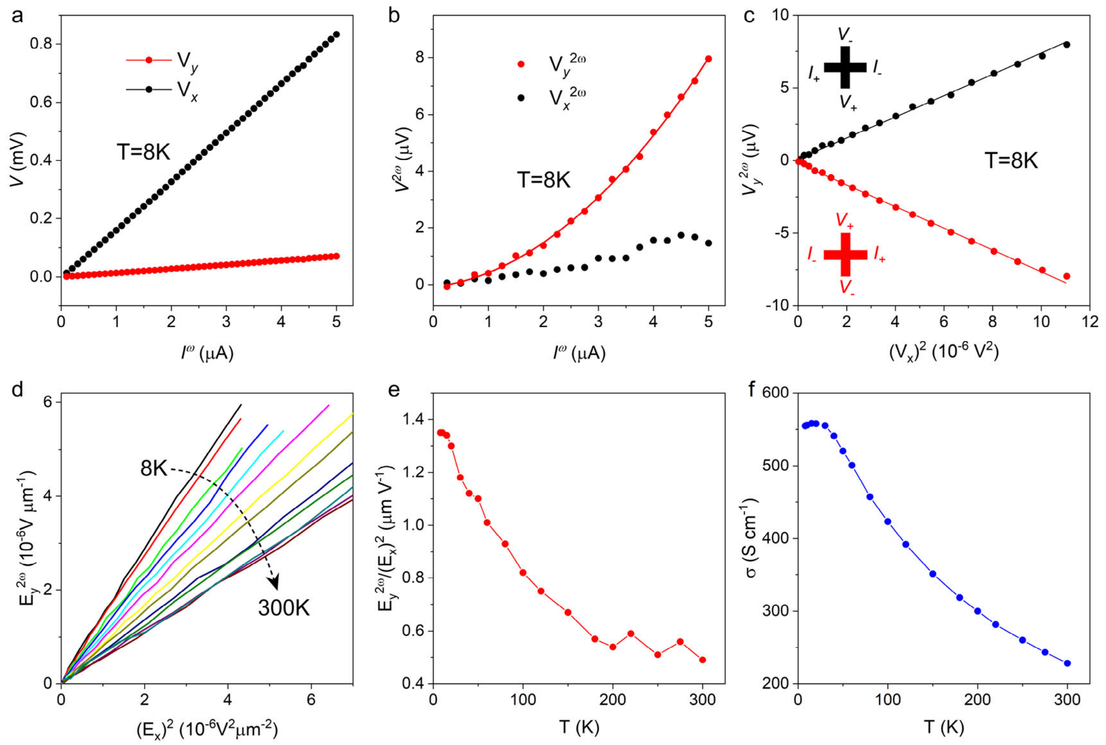

Supplementary Figure S7 | Nonlinear transport data of BiTeBr device with thickness ~ 9nm. a and **b**, First- and second-harmonic transverse and longitudinal response as a function of I^ω in BiTeBr Hall bar device at 8K, respectively. **c**, $V_y^{2\omega}$ depends linearly on the square of longitudinal voltage V_x and changes sign when the current direction and voltage probe electrodes are simultaneously reversed. **d**, $E_y^{2\omega}$ dependent of $(E_x)^2$ measured at temperature ranging from 8K to 300K. **e** and **f**, The nonlinear susceptibility $E_y^{2\omega}/(E_x)^2$ and conductivity σ as a function of temperature, respectively.

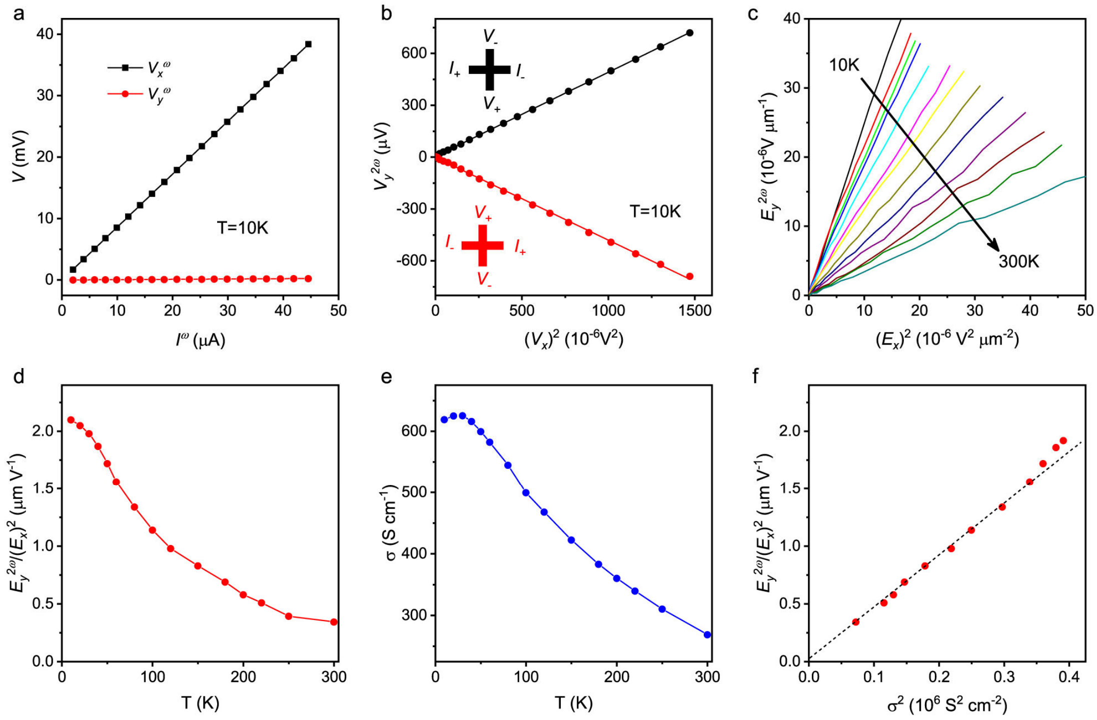

Supplementary Figure S8 | Nonlinear transport response of BiTeBr device with thickness ~ 15nm. a, V_x^ω and V_y^ω as a function of I^ω at 10K. **b**, $V_y^{2\omega}$ depends linearly on $(V_x)^2$ and changes sign when the current direction and voltage probe electrodes are simultaneously reversed. **c**, $E_y^{2\omega}$ dependent of $(E_x)^2$ measured at temperature ranging from 10 to 300K. **d** and **e**, $E_y^{2\omega}/(E_x)^2$ and σ as a function of temperature, respectively. **f**, $E_y^{2\omega}/(E_x)^2$ as a function of σ^2.

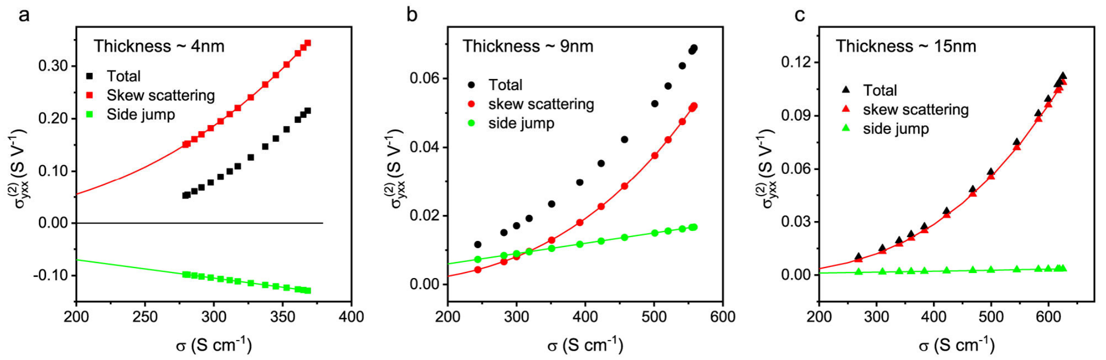

Supplementary Figure S9 | The the skew scattering and side jump contributions to second-order conductivity $\sigma_{yxx}^{(2)}$ in BiTeBr device. **a**, **b**, and **c**, The total measured $\sigma_{yxx}^{(2)}$ (black) separated into skew scattering and side jump contributions based on their σ^3 and σ dependence in 4 nm, 9 nm, and 15 nm devices, respectively.

S5. Symmetry-dependent nonlinear response in BiTeBr

A disc-shape device with 12 radially distributed electrodes was fabricated to investigate the symmetry-dependent nonlinear response in BiTeBr. A harmonic current I^{ω} was injected through two of 12 electrodes and measured the voltage at first and second harmonic frequencies in both longitudinal and transverse directions. In our experiment, the x-axis is defined as the current direction and the y-axis denotes the transverse direction to the current. The measurement was conducted at $T = 2$K.

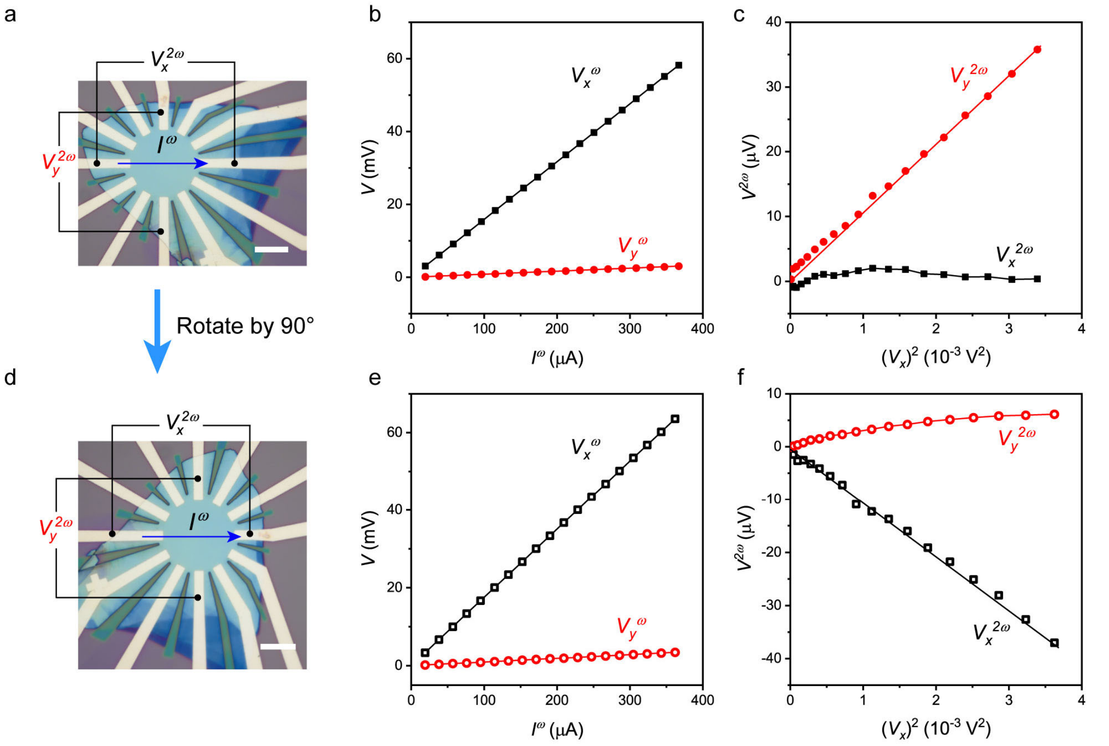

Supplementary Figure S10 | The second order nonlinear response measured at two orthogonal directions. **a**, Schematic image of the nonlinear response measured with applying current along the crystal axis. Scale bar, 10 μm. **b**, **c**, First and second harmonic longitudinal and transverse voltage as a function of alternating current I^{ω} when I^{ω} aligned along the crystal axis. **d**, **e**, **f**, Schematic image and the corresponding first and second harmonic response when rotating current direction by 90°.

The nonlinear response measured at two orthogonal directions are shown in Figure S10. Figure S10a-c illustrates the schematic and corresponding responses at first and second harmonic frequencies when the current is aligned along the crystal axis. In Figure S10b, first harmonic longitudinal voltage (V_x^{ω}) exhibits a linear increase with I^{ω}, while the transverse voltage (V_y^{ω}) remains minimal, indicating good ohmic contact and negligible

electrode misalignment. In Figure S10c, the nonlinear transverse response ($V_y^{2\omega}$) scales linearly with the square of V_x, while the longitudinal response ($V_x^{2\omega}$) shows negligible and irregular response. When we rotate the direction of I^{ω} by 90°, as depicted in Figure S10d-f, the $V_x^{2\omega}$ scales linearly with $(V_x)^2$ with a negative sign, while $V_y^{2\omega}$ displays small and negligible response. The value of $V_y^{2\omega}$ and $V_x^{2\omega}$ measured at two orthogonal directions is almost equally. These results comply with the C_{3v} symmetry of BiTeBr. Under C_{3v} symmetry, $\sigma_{yxx} = -\sigma_{yyy}$ is nonzero, and $\sigma_{xyy} = -\sigma_{xxx} = 0$, here x aligns along with the crystal axis and y is the direction perpendicular to crystal axis.

A completely symmetry-dependent characteristics of nonlinear response of BiTeBr are shown in Figure S11. A harmonic current I^{ω} was applied with an injection angle θ to the crystal axis, and we measured the nonlinear response in both longitudinal and transverse directions. Both the longitudinal and transverse nonlinear response exhibit a three-fold symmetry, complying with the expected C_{3v} symmetry.

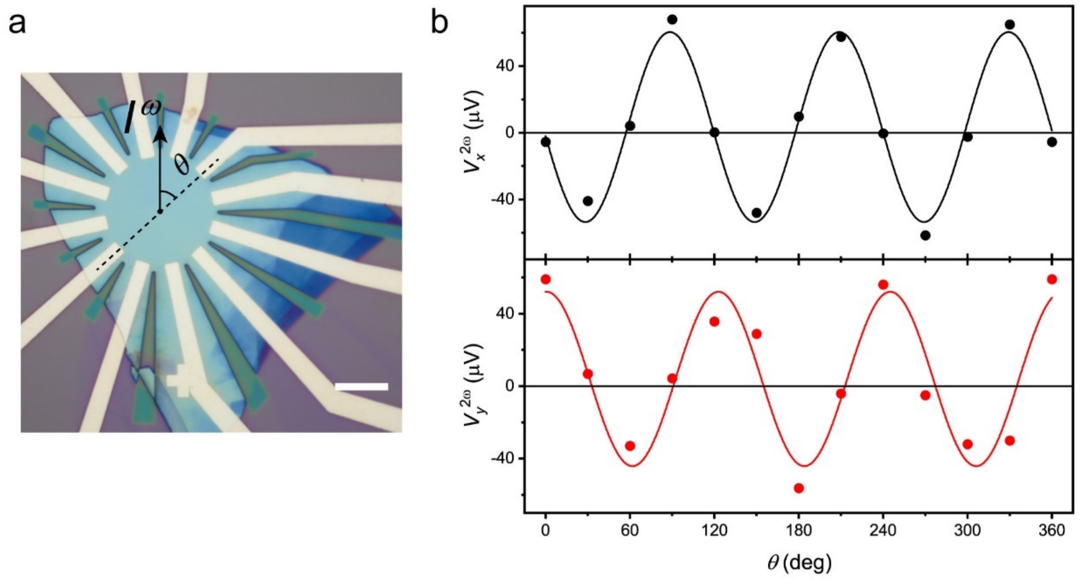

Supplementary Figure S11 | The angle dependence of the second order nonlinear response of BiTeBr in both longitudinal and transverse directions. **a**, Optical image of the 25nm-thick BiTeBr device. The electrode is deliberately aligned with the crystal axis, as marked with the dashed line. Scale bar, 10 μm. **b**, The second order nonlinear longitudinal and transverse voltages as a function of current injection angle θ.

S6. Fitting the experimental nonlinear data to the theoretically derived equation

To verify whether the eq. (5) derived from theory in the main text can quantitatively matching with our experimental data, we fitted our experimental data based on eq. (5). Since $\frac{E_y^{(2)}}{(E_x)^2} = \frac{\sigma_{yxx}^{(2)}}{\sigma}$, the second order nonlinear conductivity ($\sigma_{yxx}^{(2)}$) can be obtained from the experimental nonlinear susceptibility $\frac{E_y^{(2)}}{(E_x)^2}$ and conductivity σ. The nonlinear conductivity as a function of temperature for different thickness BiTeBr devices are shown in Figure S10**a-c**.

To fit with eq. (5) in the main text, a constant *C₀* was brought in for ease of fitting, $\frac{\sigma_{yxx}^{(2)}(T)}{\sigma_{yxx}^{(2)}(0)} = C_0(1+aT)^{-3}\tanh\frac{\epsilon_R}{2k_\mathrm{B}T}$. For the 4 nm device, we selected $\sigma_{yxx}^{(2)}(0)$ as 0.22 SV^{-1} to determine $\sigma_{yxx}^{(2)}(\mathrm{T})/\sigma_{yxx}^{(2)}(0)$. This ratio, as a function of temperature for the 4 nm device, is illustrated in Figure S10d with red scattered circles. By fitting the data, we obtain $a = 3.5 \times 10^{-3} \pm 5 \times 10^{-3}\ K^{-1}$, $\epsilon_R = 0.05 \pm 0.009$ eV, $C_0 = 1.04 \pm 0.1$, with the fitting curve presented with red solid line in Figure S10d. The experimental data $\sigma_{yxx}^{(2)}(\mathrm{T})/\sigma_{yxx}^{(2)}(0)$ fits well with the theory eq. (5), implying it can indeed provide a quantitative description of the Rashba splitting's role in skew scattering-induced nonlinear response. The slight discrepancy between the fitted ϵ_R (50eV) and the Rashba splitting energy in BiTeBr (42 eV) might arise surface effects contributing to the nonlinear response. Furthermore, the larger fitted value of 'a' ($3.5 \times 10^{-3}\ K^{-1}$) compared to the one derived from the temperature dependence of conductivity ($a \sim 1.1 \times 10^{-3}\ K^{-1}$) may be attributed to influences of surface state and device disorder.

Adopting with the same method, the experimental data $\sigma_{yxx}^{(2)}(\mathrm{T})/\sigma_{yxx}^{(2)}(0)$ and its fitting curve with eq. (5) for 9 nm and 15 nm thick devices are shown in Figure R10e, f. The fitted value of 'a' increase with thickness, while the ϵ_R decrease, indicating the influence of the surface effect is reducing.

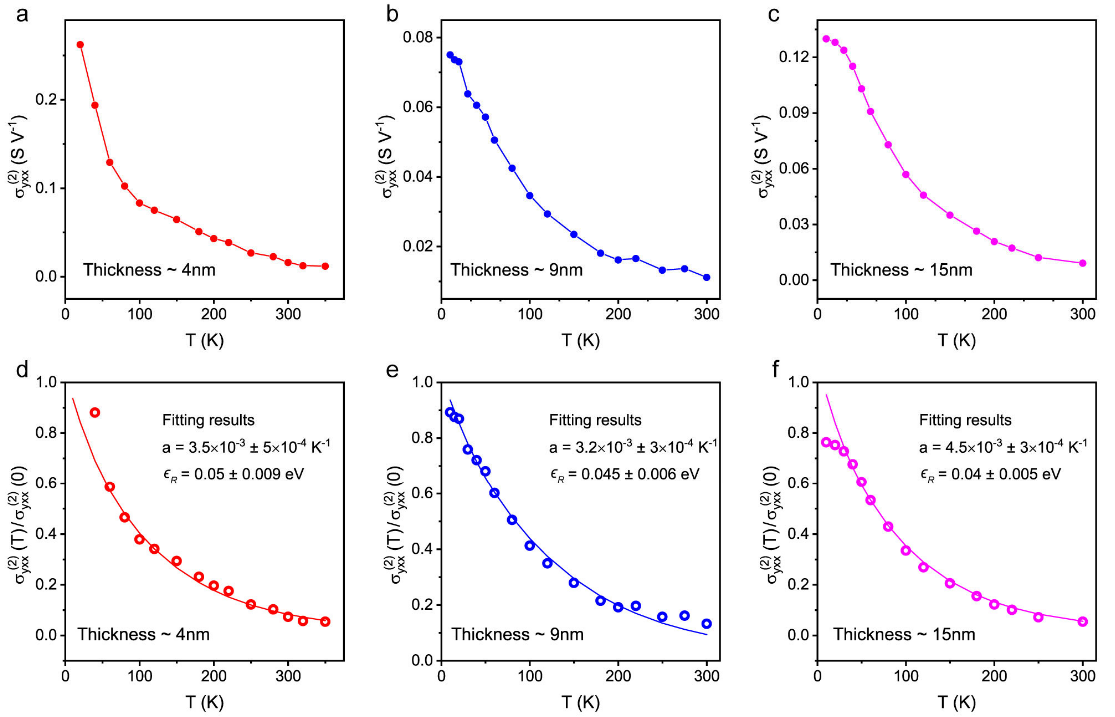

Supplementary Figure S12 | The experimental second order nonlinear conductivity ($\sigma_{yxx}^{(2)}$) and its corresponding theoretical fitting for different thickness BiTeBr device. a and d, Experimental $\sigma_{yxx}^{(2)}$ in 4 nm device and its corresponding fitting of $\sigma_{yxx}^{(2)}(\mathrm{T})/\sigma_{yxx}^{(2)}(0)$ using eq. (5) in the main text. b and e, Experimental $\sigma_{yxx}^{(2)}$ in 9 nm device and its corresponding theoretical fitting of $\sigma_{yxx}^{(2)}(\mathrm{T})/\sigma_{yxx}^{(2)}(0)$. c and f, Experimental $\sigma_{yxx}^{(2)}$ in 15 nm device and its corresponding theoretical fitting of $\sigma_{yxx}^{(2)}(\mathrm{T})/\sigma_{yxx}^{(2)}(0)$.

S7. Additional wireless radiofrequency rectification data

Figure S13b shows the rectified DC voltage as a function of wireless RF power for frequencies of 0.20, 0.34, 0.40, and 0.68 GHz. Figure S13c demonstrates the variation of the rectified DC voltage (V^{DC}) with RF frequency, by combining the data obtained at frequencies of 1,0, 2.4, 3.8, and 5.9 GHz. These results indicate that the NLHE of BiTeBr device can effectively rectify wireless RF over a broad band (0.2 to 6 GHz) with the rectified voltage decreasing as the RF frequency increases.

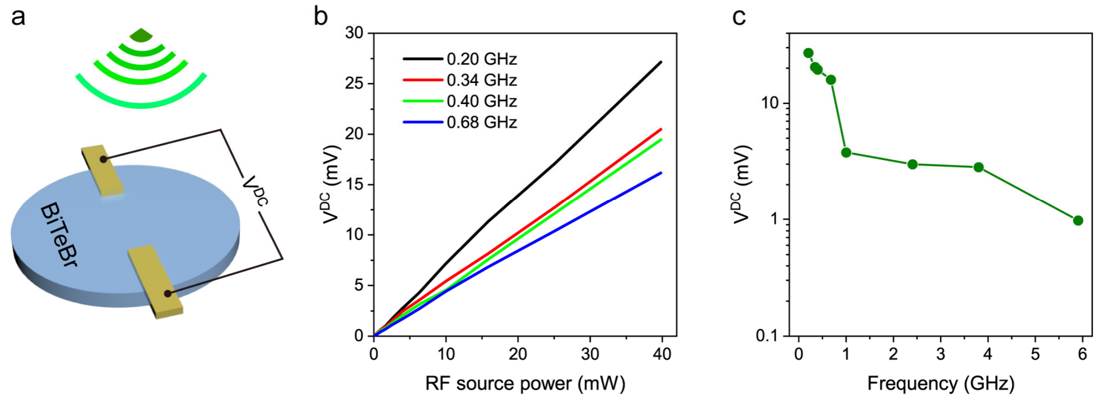

Supplementary Figure S13 | Wireless RF rectification measured on 22 nm-thick BiTeBr device at room temperature. a, Schematic of the rectification measurement setup. **b**, The output rectified DC voltage as a function of wireless RF power under frequencies of 0.20, 0.34, 0.40, and 0.68 GHz. **c**, The output DC signal V^{DC} as a function of RF frequency. The RF power was 16dBm (~ 40mW).

References

1. Nagaosa, N., Sinova, J., Onoda, S., MacDonald, A. H., and Ong, N. P. Anomalous Hall effect. *Rev. Mod. Phys*. **82**, 1539 (2010).

2. He, P. *et al*. Quantum frequency doubling in the topological insulator Bi_2Se_3. *Nat. Commun*. **12**, 698 (2021).

3. Hsieh, D. *et al*. Nonlinear optical probe of tunable surface electrons on a topological insulator. *Phys. Rev. Lett*. **106**, 057401 (2011).

4. Hsieh, D. *et al*. Selective probing of photoinduced charge and spin dynamics in the bulk and surface of a topological insulator. *Phys. Rev. Lett*. **107**, 077401 (2011).

5. Hsu, W.-T. *et al*. Second harmonic generation from artificially stacked transition metal dichalcogenide twisted bilayers. *ACS Nano* **8**, 3, 2951-2958 (2014).

6. Fonseca, J. *et al*. Anomalous second harmonic generation from atomically thin $MnBi_2Te_4$. *Nano Lett*. **22**, 10134-10139 (2022).
